# Injury factors alter miRNAs profiles of exosomes derived from islets and circulation

**DOI:** 10.18632/aging.101689

**Published:** 2018-12-14

**Authors:** Qi Fu, Hemin Jiang, Zibin Wang, Xingyun Wang, Heng Chen, Ziyang Shen, Lei Xiao, Xirong Guo, Tao Yang

**Affiliations:** 1 Department of Endocrinology and Metabolism, The First Affiliated Hospital of Nanjing Medical University, Nanjing, Jiangsu 210029, China; 2 Analysis Center, Nanjing Medical University, Nanjing, Jiangsu 211166, China; 3 Nanjing Maternal and Child Health Medical Institute, Obstetrics and Gynecology Hospital Affiliated to Nanjing Medical University, Nanjing, Jiangsu 210004, China; * Equal contribution

**Keywords:** islets, exosomes, miRNA, cellular injury, biomarker

## Abstract

Islets damage is a major abnormality underling diabetes. Recent studies suggested the value of exosomes in diagnosis. This study aimed to investigate the impact of injury factors on the miRNA profiles of islet exosomes and determine whether circulating exosomal miRNAs is suitable as biomarkers of islets damage. Islets were isolated from ICR mice and induced injury in vitro by mixed cytokines (Tumor Necrosis Factor-α, Interleukin -1β and Interferon-γ) or streptozotocin (STZ), and exosomes were derived from the cultural supernatant. Using miRNA microarray analysis, we found 22 and 11 differentially expressed miRNAs in islet exosomes of STZ and cytokines treatment, respectively, including 6 miRNAs as the intersection of two injured conditions. Thereinto, mmu-miR-375-3p and mmu-miR-129-5p could be validated by qRT-PCR. Then, Serum exosomes were isolated from STZ injected mice and subjects with various glucose metabolism states and diabetic duration. qRT-PCR demonstrated exosomal mmu-miR-375-3p dramatically increased in serum of STZ treated mouse prior to the disturbance of blood glucose and insulin. In human serum exosomes, hsa-miR-375-3p was elevated in new-onset diabetes patients. Overall, our results suggest that injury factors changed miRNA profiles of exosomes derived from islets and exosomal miR-375-3p showed promising potential as a biomarker of islets damage.

## Introduction

Pancreatic islets destruction and cell death is major pathophysiologic abnormalities underling both type 1 diabetes (T1DM) and type 2 diabetes (T2DM), and absolute or relative defects of β cell insulin secretion are characterized by almost all forms of diabetes [1]. Because of the heterogeneous etiologies of diabetes, the rate and extent of islets destruction are diverse [2]. Timely detection and assessment of islet destruction facilitates prevention and intervention to protect islet β cell function.

Exosomes as one sort of extracellular vesicles (EVs) are secreted into extracellular space by most cell types and found in many bodily fluids. Exosomes are composed of a lipid bilayer and contained soluble and membrane-bound protein, genomic DNA, RNA (such as mRNA, miRNAs, and other small RNAs), lipids and metabolites derived from the parent cells [3,4]. By virtue of their various cargoes, exosomes are recognized as essential conveyers of cellular information and participate pleiotropic effects and biological functions in multicellular organisms [5–7]. In consequence, exosomes exhibit promising roles as disease biomarkers and gained immense interests in recent years [8,9]. MiRNA as a member of small noncoding RNA (ncRNA) family is abundant in exosomes. Furthermore, overwhelming evidences demonstrated that exosomes could accomplish miRNA processing outside mammalian cells [10,11].

Therefore, the aim of this study was to investigate the miRNA profiles of exosomes isolated from islets suffering injury factors. Cytokine is one of the most common risk factors causing islet β cells injury. A series of studies indicated that tumor necrosis factor α (TNFα), interleukin-1 β (IL-1β) and Interferon γ (IFNγ) cytokines cocktail could result in endoplasmic reticulum stress (ERS) and apoptosis in rodent and human β cells. Moreover, in vivo and in vitro animal experiments, streptozocin (STZ) is often used to induce diabetes models, which is islet β cell specific damaging agent [12,13]. In order to ensure the reliability of the results in our study, both cytokines and STZ were used to induce islet injury in vitro, respectively. The miRNA expression profiles of exosomes derived from islets suffering different injury factors were screened using microarray assay, and the intersection of the two treatments were selected as further verification indices. Next, to explored whether the screened exosomal miRNAs in vitro could be detected in circulation and considered as novel biomarkers of islets damage, circulating exosomes were isolated from diabetic mouse models and human patients and analyzed in terms of miRNAs expression. The flow diagram of this experiment was shown in Supplementary Figure S1.

## RESULTS

### Islets injury induced by cytokines or STZ in vitro

**Figure 1:**
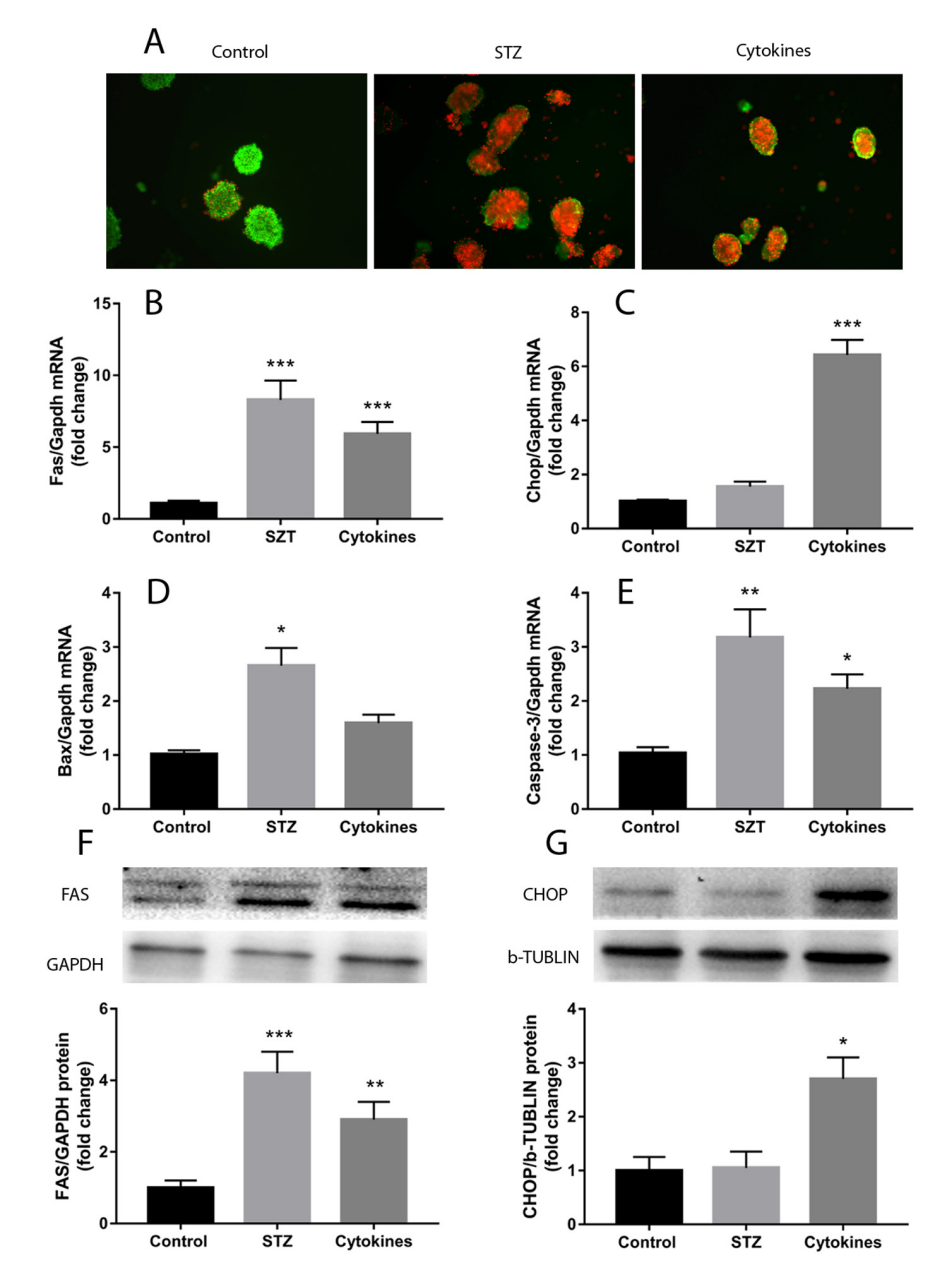
**Islets injury induced by STZ or cytokines in vitro.** Islets isolated from ICR mice were treated with STZ or TNFα, IL-1β and IFNγ cytokines cocktail. (**A**) AO/PI staining of islets with STZ, cytokines and control. (**B**-**E**) qRT-PCR measured the expression of apoptosis and endoplasmic reticulum stress related genes (*Fas*, *Chop*, *Bax*, *Caspase-3*) in islets after different treatment. (**F**, **G**) FAS and CHOP quantification by western blot. The data are presented as mean±SEM of three independent experiments. *, **, *** denote a significant difference between STZ or cytokines treatment and control (* *P*<0.05, ** *P*<0.01, *** *P*<0.001).

Treatment with STZ or TNFα, IL-1β and IFNγ cytokines cocktail induced noticeable injury of mouse islets. After 24 hours incubation, islets of control group showed normal morphology and reasonable viability, whereas STZ and cytokines led to cell death and islets disintegration showed in AO/PI staining (Figure 1A). Both STZ and cytokines cocktail remarkably increased the expression of apoptosis related genes, including *Caspase-3* and *Fas* (Figure 1B and E). The ERS marker C/EBP homologous protein (*Chop*) gene was significantly upregulated only in triple cytokines treated islets, meanwhile, the Bcl-2 associated X protein (*Bax*) gene expression was exclusively increased in STZ treatment (Figure 1C and D). Western blot tests further confirmed the upregulation of FAS and CHOP (Figure 1F-G).

### Isolation and characterization of exosomes

**Figure 2:**
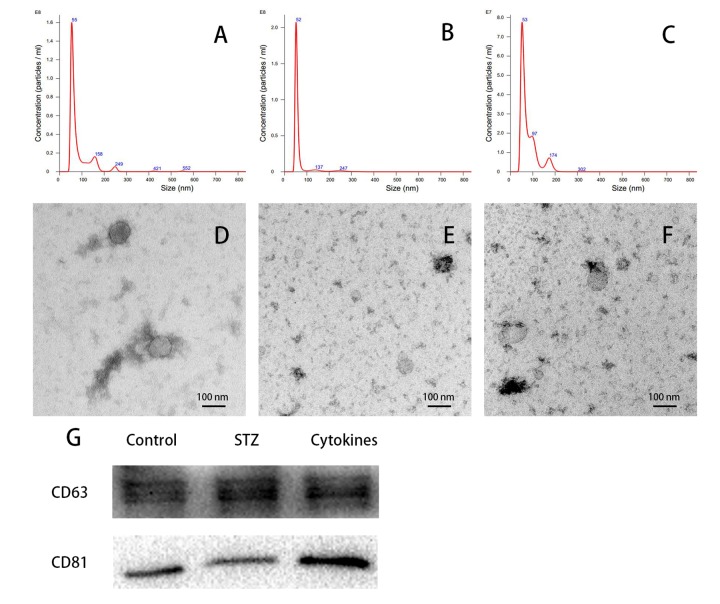
**Characterization of islets-released exosomes.** Exosomes derived from islets culture medium were characterized by NTA, TEM and western blot. (**A**-**C**) Particle and size distribution of analyzed derived from control, STZ and cytokines treated mouse islets were mostly within the normal range for exosome size (30-150 nm in diameter). (**D**-**F**) TEM of negatively staining showed the morphology of control, STZ and cytokines treated islet exosomes respectively. (**G**) Western blot analysis of CD63 and CD81 expression in exosomes.

Nanoparticle tracking analysis (NTA) showed the size of most isolated particles from culture medium of control, STZ and cytokines treated mouse islets were within the normal range of exosome size (30-150 nm in diameter, Figure 2A-C). Transmission electron microscopy (TEM) of negatively stained exosomes identified spherical vesicles, and the size was in accordance with NTA results (Figure 2D-F). Western blot analysis verified the expression of exosomal markers CD63 and CD81 in mouse islets exosomes, which is consistent with previously report (Figure 2G).

### MiRNAs profiles of exosomes derived from islets suffering different injury factors in vitro

**Figure 3:**
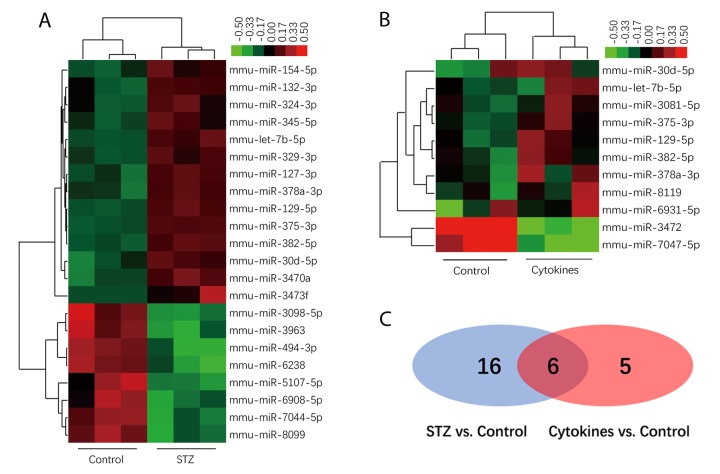
**MiRNAs profiles of exosomes derived from islets.** Heatmap of the normalized expression levels of miRNAs diﬀerentially expressed in exosome of STZ (**A**) and cytokines (**B**) treated islets compared with control (x axis clustering showed different treatments). (**C**) Venn diagram show 6 differentially expressed miRNAs both in STZ and cytokines treated group compared with controls (shown in the overlap area).

**Figure 4:**
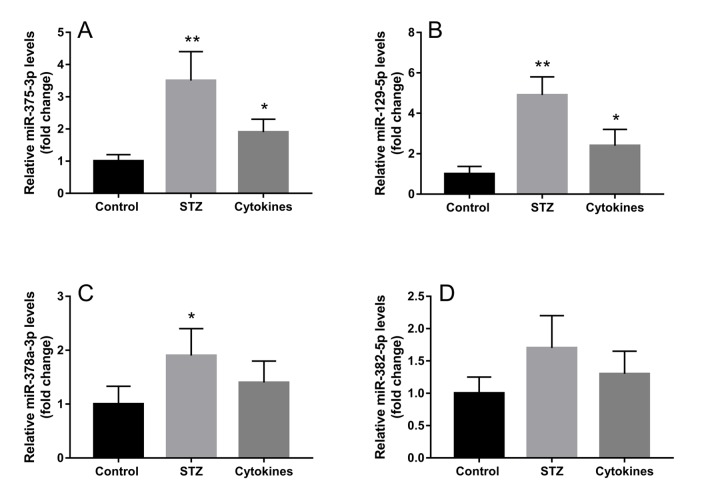
**Validation of exosomal miRNAs detected by microarray.** Four miRNAs with top high FC values (FC>1.5 in both STZ and cytokines treated groups) miR-375-3p (**A**), miR-129-5p (**B**), miR-378a-3p (**C**), miR-382-5p (**D**) were further verified by qRT-PCR in three independent samples. The data are presented as mean±SEM. (* *P*<0.05, ** *P*<0.01 compared with control).

The miRNA microarray assay investigated miRNA expression profiles of exosomes derived from islets suffering different injury factors in vitro. Each group including three independent samples of islets exosomes, and every one sample of exosomes was isolated from islets of 10-15 ICR mice. Among the 1881 mature miRNAs in microarray, the detection rate ranged from 12.71% to 26.74%. As shown in the heat map (Figures 3A and B), the differentially expressed miRNAs in exosomes of STZ and cytokine-treated islets identified by microarray were not identical. Compared with control group, there were 22 and 11 differentially expressed miRNAs (*P*<0.05) in STZ and cytokines treated group respectively. Venn diagram (Figure 3C) showed that 6 differentially expressed miRNAs mmu-let-7b-5p, mmu-miR-30d-5p, mmu-miR-129-5p, mmu-miR-375-3p, mmu-miR-378a-3p, mmu-miR-382-5p were found both in STZ and cytokines treated group, which were all up-regulated compared with control. To assess the potential of these miRNAs as biomarkers for islet injury, 4 miRNAs (FC>1.5 in both STZ and cytokines treated group) including mmu-miR-129-5p, mmu-miR-375-3p, mmu-miR-378a-3p, mmu-miR-382-5p were selected for further validation. The change levels of these miRNAs detected by microarray were verified by qRT-PCR in independent samples (Figure 4). As shown by the qRT-PCR results, mmu-miR-375-3p and mmu-miR-129-5p were significantly higher in both STZ and cytokines group, meanwhile, mmu-miR-378a-3p was only differentially expressed in STZ group. On the other hand, mmu-miR-382-5p was not verified in both STZ and cytokines group.

### Expression of screened miRNAs in serum exosomes of STZ treated mice

**Figure 5:**
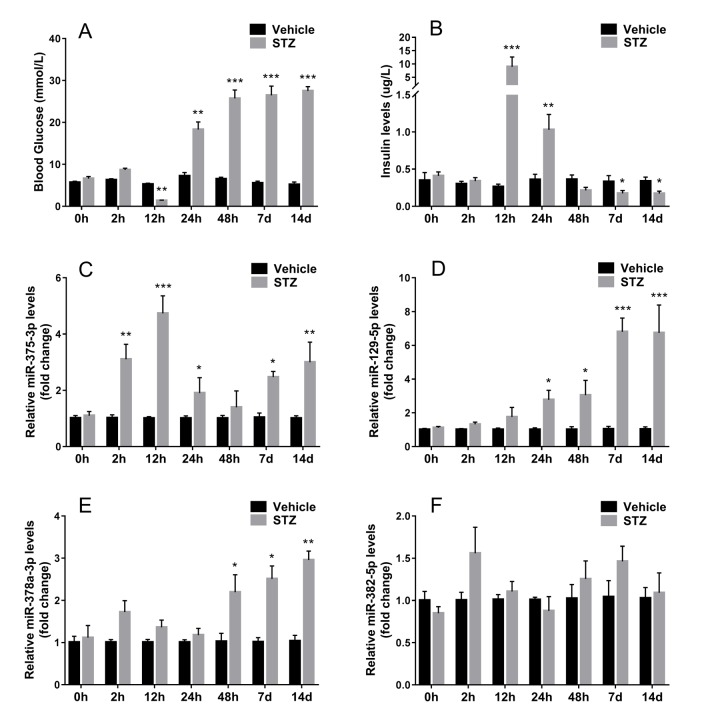
**Expression of screened miRNAs in serum exosomes of STZ treated mice.** Serum was sampled from ICR mice at different time after STZ injection. Fasting tail blood glucose (**A**) and serum insulin (**B**) levels were measured. The expression of 4 serum exosomal miRNAs selected from microarray miR-375-3p (**C**), miR-129-5p (**D**), miR-378a-3p (**E**), miR-382-5p (**F**) were analyzed using qRT-PCR. Values represent means ± SEM (n≥9 mice of each time point). (* *P*<0.05, ** *P*<0.01, *** *P*<0.001 compared with sodium citrate vehicles).

To access whether exosomal miRNA could be detected in serum and changes synchronously when islet β cells were injured in vivo, we injected STZ intraperitoneally into ICR mice, then isolated serum exosomes and analyzed exosomal miRNA by qRT-PCR. After STZ administration, blood glucose decreased at 12 hours and then increased gradually (Figure 5A). On the other hand, serum insulin levels reached the peak after 12 hours of STZ injection and declined soon afterwards (Figure 5B), which indicated drastic insulin release during β cell death induced by STZ. When examining the levels of the aforementioned 4 screened miRNAs in serum exosomes of STZ treated mice, mmu-miR-375-3p showed significant increase at 2 hours after STZ administration compared with control, however, blood glucose and serum insulin levels did not change at this moment. Then mmu-miR-375-3p peaked to 4.7-fold compared with controls at 12 hours after STZ injection and gradually decreased with time until 48 hours. It is noteworthy that exosomal mmu-miR-375-3p levels rose again at 7 and 14 days after injcetion (Figure 5C). Levels of serum exosomal mmu-miR-129-5p started to escalate from 24 hours to 6.7-fold at 14 days, which is in accordance with the tendency of blood glucose (Figure 5D). The increase of mmu-miR-378a-3p levels was later and slimmer than mmu-miR-129-5p (Figure 5E). There was no significant change of serum exosomal mmu-miR-382-5p during the 14 days observation period after STZ treatment(Figure 5F).

### Serum exosomal miRNA-375-3p and miRNA-129-5p expression in T1DM and T2DM patients with different β cell function

**Figure 6:**
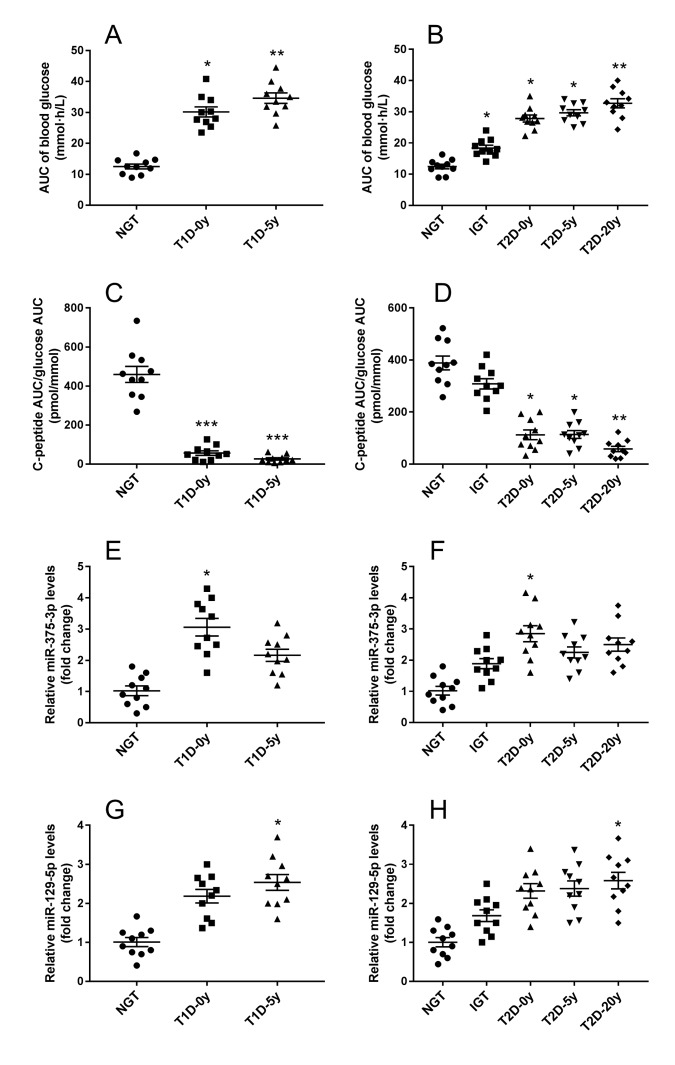
**Serum exosomal miRNA-375 and miRNA-129 expression in T1DM and T2DM patients with different β cell function.** After standard mixed meal tolerance tests, the area under curve (AUC) of plasma glucose were measured (**A**), and the ratio of C-peptide AUC to glucose AUC were calculated to evaluate islet β cell function (**B**). Serum exosomal miRNA-375 (**E**, **F**) and miRNA-129 (**G**, **H**) expression in T1DM and T2DM patients with diverse diabetes course were detected by qRT-PCR. All measurements are mean ± SEM (n≥10 subjects of each group) (* *P*<0.05, ** *P*<0.01, *** *P*<0.001 compared with NGT.)

Whether T1DM or T2DM, islet cell damage is involved in the development and progression of the disease. With the diabetes course being longer, the decrease in insulin secretion capacity of islet β cells indirectly reflects the degree and speed of islet damage. As exosomal mmu-miR-375-3p and mmu-miR-129-5p markedly changed in serum of STZ treated mice, these 2 miRNAs were tested in diabetes patients with different diabetes course and control subjects. Both in T1DM and T2DM patients, area under the curve (AUC) of blood glucose showed an increased tendency along with the diabetes course extension (Figure 6A and B), conversely, the β cell function (C-peptide AUC/ glucose AUC) gradually deceased with the time (Figure 6C and D). Interestingly, although the level of serum exosomal hsa-miR-375-3p was elevated seemingly in all impaired glucose tolerance (IGT) and diabetes patients, it was only significantly increased in newly diagnosed T1DM (3.1±0.3 fold change) and T2DM patients (2.8±0.3 fold change) (Figure 6E and F). These results may be an indicative of serious damage of β cells during the onset of diabetes. The serum exosomal hsa-miR-129-5p appeared significantly upregulated in T1DM patients with 5 years course (2.5±0.2 fold change) and T2DM with 20 years course (2.6±0.3 fold) (Figure 6G and H).

## DISCUSSION

Islet of Langerhans is one of the most abustle organs in whole body, responsible for maintaining steady blood glucose level. Because of the intensive workload, islets rich in β cells are susceptible to sorts of stressors, such as inflammation, ERS, oxidative stress and amyloid stress, which ultimately lead to β cell death and loss of islet mass [14–16]. To detect and monitor islets injury, several groups have evaluated the profiles of free nucleic acids and protein from plasma or bodily fluids as biomarker. Exosomes with cargoes of stable and tissue-specific RNA and proteomic signature profiles reflect the conditional states of their original cells. Previous studies indicated that miRNAs carried by exosomes may do not simply identify with the intracellular content, some miRNAs are preferentially released in exosomes [17,18]. Although the mechanisms regulating the cargoes of miRNAs in exosomes remain unclear, the miRNA profiles might be used as biomarkers for disease and health status of specific organs. In agreement with this standpoint, our study demonstrated that different injury factors have diverse impact on miRNA contents of exosomes released by mouse islets, meanwhile, several miRNAs coexisted in islet exosomes under different damaging conditions.

Both pro-inflammatory cytokines such as TNFα, IL-1β and IFN-γ cocktails and STZ are often used to induce islet β cell injury in vitro and in vivo, however, the mechanism whereby they lead to β damage was not of the same. Consistent with the results of this study, previous studies confirmed TNFα, IL-1β and IFN-γ cocktails mainly activate ERS pathway, nevertheless STZ induces toxicity mainly by inducing ROS and nitric oxide production [19,20]. Therefore, the effects of these two injury factors on the profiles of miRNAs in islet exosomes are also different, however, there is intersection between the two conditions. The coexisting exosomal miRNAs under various damaging conditions are more likely to be biomarkers of islet injury. Our data showed that mmu-miR-375-3p, mmu-miR-129-5p were enriched in exosomes both derived from both cytokines and STZ treated mouse islets, which were confirmed by microarray and qRT-PCR. In vivo experiments with STZ-injected mice, circulatory exosomal mmu-miR-375-3p increased preceding the disturbance of blood glucose and serum insulin. After peaking at 12 hours of STZ injection, serum exosomal mmu-miR-375-3p gradually decreased in accord with the loss of islet β cells. In addition, the sequence of miR-375-3p is completely conserved between mice and humans (shown in Supplementary Table S1). In both T1DM and T2DM patients, serum exosomal hsa-miR-375-3p was significantly elevated in newly diagnosed patients. At the onset of diabetes most patients possess a considerable amounts of residual islets β cell being suffering damage [21]. These observations indicated the potential utility of exosomal miR-375-3p detecting islets injury.

In our study, one phenomenon worthy of attention is that exosomal mmu-miR-375-3p of serum from STZ treated mouse went up again during the late stage after STZ injection, while the blood glucose increased remarkably. In addition, the changing trend of serum exosomal mmu-miR-129-5p was in keeping with blood glucose. This phenomenon cannot be attributed to islets injury and β cells death, because most islets were eliminated by β cell toxic STZ at the moment. Another issue to be aware of is that there was discordance in the miRNAs expression of exosomes between islets culture media and serum, such as mmu-miR-378a-3p and mmu-miR-382-5p in our results. This divergence reflected that whole circulatory exosome analysis was not sufficient and accurate adequately to identify islets injury, as many tissues contribute to the total plasma exosome pool [22]. Recent study demonstrated that adipose tissue derived exosomes are one major source of circulating miRNAs [23]. Although miR-375-3p is abundantly expressed in pancreatic islets, on account of the mini quantity of islets in whole body, the test of circulating exosomal miR-375-3p had high noise-to-signal ratio.

Some previous studies showed free circulating miR-375-3p was associated with β cell death and could be used to predict hyperglycemia in mouse models of T1DM [24]. Subsequent researches also detected elevated circulating miR-375-3p in the plasma of T1DM and T2DM subjects [25,26]. Even so, free circulating miRNAs as biomarkers were not strictly specific and accurate, and one kind of free circulating miRNAs may exhibit similar changes in more than one disease or pathological conditions. This drawback of free circulating miRNA as biomarker was intractable so far. Although the whole circulatory exosome analysis has similar problem, the conception of isolating islet-specific exosomes may settle this matter. A recent research on islets transplantation successfully collected and quantified exosomes of transplanted islets from recipient plasma using anti-HLA antibody conjugated beads, and also identified distinct changes in proteomic and miRNA profiles of these exosomes, which demonstrated attractive potential to monitor immunologic rejection [27]. Another interesting study found that rat and human pancreatic islets release β cell specific autoantigens GAD65, IA-2 and proinsulin in exosomes [28]. Furthermore, β cell specific surface marker FXYD2 isoforms γa and γb were reported to be co-expressed in exosomes of transplanted islets. All these findings sketched out a promising blueprint to purify islet-specific exosomes from circulation, and our research provided the characterizing data of exosomal miRNAs associated with islets injury.

As containing proteins and nucleic acids of parental cells that can be transferred to recipient cells, exosomes mediate cellular communication in diabetes and metabolic disease. Accumulating evidences supported the pathophysiologic function of exosomes released by pancreatic islets. It has been reported that exosomes participate in the crosstalk between islet β cells and endothelial cells or lymphocytes [29,30]. Moreover, islet β cells secrete exosomes can be horizontally transferred to neighboring cells. Exposure of pro-inflammatory cytokines modified the profiles of exosomal miRNAs and impaired survival of recipient β cells [31]. Substantial studies confirmed that miR-375-3p is involved in β cell proliferation and secretion function of β and α-cells [32]. The present study showed that injury factors alter miRNAs profiles of islet exosomes including miR-375-3p level, which added further weight to the theory of horizontal communication between islet cells via exosomes.

In conclusion, our results demonstrated injury factors cytokines and STZ have noticeable impact on the miRNA profiles of exosomes derived from islets. Exosomal miR-375-3p dramatically increased in circulation of STZ treated mouse prior to hyperglycemia and in new-onset diabetes patients, which showed promising potential as a biomarker of islets damage. However, further studies would be necessary to develop new techniques to purify islet-specific exosomes from circulation to improve the accuracy.

## MATERIALS AND METHODS

### Animals

Islets were isolated from eight to ten weeks old male ICR mice which were purchased from the Model Animal Research Center of Nanjing University. All experimental procedures of animals were approved by the Medicine Animal Care Committee of Nanjing Medical University. To establish mouse models of islets injury in vivo, a subset of ICR mice were injected of STZ (Sigma Aldrich, Saint Louis, USA; dissolved in 0.1M sodium citrate, pH 4.5) intraperitoneally at a dose of 180 mg/kg, meanwhile control mice received vehicle injections (0.1M sodium citrate, pH 4.5) correspondingly [33]. Blood samples were drawn from orbits and stood 1 hour for coagulation, followed by centrifugation at 3000 rpm, 30 minutes for serum. Serum insulin levels were measured using Mercodia Mouse Insulin ELISA kit (Mercodia AB, Uppsala, Sweden). Tail blood glucose was measured after 4-hour morning fasting using MEDISAFE MINI (Terumo, Tokyo, Japan).

### Study population

Subjects with various glucose metabolic states (including T1DM, T2DM, IGT and healthy control) and diabetic duration were recruited through poster. Diabetes mellitus and IGT were diagnosed according to the criteria worked out by World Health Organization (WHO) in 1999 (diabetes: fasting blood glucose >7.0 mmol/L and/or 2h postprandial blood glucose ≥11.1 mmol/L; IGT: 7.8 mmol/L < 2h postprandial blood glucose <11.1 mmol/L). T2DM patients were grouped into newly diagnosis (T2D-0y), 5 years course (T2D-5y) and 20 years course (T2D-20y), meanwhile, T1DM patients were grouped into newly diagnosis (T1D-0y), 5 years course (T1D-5y). Each group of this study included at least 10 subjects. The average age of T1DM and corresponding control (normal glucose tolerance, NGT) were 26.3±5.7 and 24.9±3.3 (mean±SD), respectively. Meanwhile, subjects of T2DM, IGT and corresponding NGT have average age of 52.8±7.2, 48.1±6.1 and 45.6±5.9 (mean±SD), respectively. All participants underwent a standard mixed meal tolerance test (MMTT) after overnight fasting. Venous blood samples were collected from intermedian cubital vein at 0 min, 30 min, 90 min and 120 min of MMTT for C-peptide and glucose analysis. After blood coagulated, serum was pipetted following centrifugation at 3000 rpm for 30 minutes. The ratio of C-peptide AUC to glucose AUC was calculated to evaluate islet β cell function. Each subject provided written informed consent in this study. All procedures were conducted in accordance with the principles of the Declaration of Helsinki.

### Mouse islets isolation and culture

After being anesthetized with chloral hydrate (50ul/10g of 0.1g/ml solution), the common bile duct of mice was cannulated and injected of collagenase P (1mg/ml; Roche Diagnostics, Mannheim, Germany) in cold Hanks’ balanced salt solution (HBSS). The distended pancreas was excised and digested at 37°C for 10 min, and then shaken by hand for 10 s until the solution appears homogeneous. The digested tissues were filtered through a 600 μm mesh and washed by cold HBSS with 10% fetal bovine serum (FBS). Islets were purified using the Histopaque purification method (Histopaque 11191 and 10771, Sigma Aldrich, Saint Louis, USA) [34]. Freshly isolated islets were plated equivalently on sterile six-well plates and cultured in RPMI-1640 medium containing 10% FBS depleted of exosomes, 1% (v/v) antibiotics (100 U/ml penicillin: 0.1 mg/ml streptomycin, Sigma Aldrich, Saint Louis, USA) and 11.1 mM glucose at 37°C, 5% CO_2_. To induce islets injury in vitro, islets were treated with three cytokines cocktail (TNFα 10 ng/ml, IL-1β 5 ng/ml and IFNγ 100 ng/ml, R&D Systems, Minneapolis, USA) or 0.5 mM STZ (in 0.1M sodium citrate, pH 4.5) for 24 hours [35]. Islet survival was evaluated by acridine orange/propidium iodide (AO/PI) staining.

### Exosome isolation

Islets exosomes were isolated with ExoQuick-TC Exosome Precipitation Solution (SBI, Palo Alto, USA). After islets were incubated in RPMI-1640 medium with cytokines cocktail or STZ for 24 hours, the media was collected and centrifuged at 3000g for 15 min at 4°C. Then, the supernatant was mixed with appropriate volume of ExoQuick-TC and refrigerated overnight at 4°C followed by a centrifugation step of 1500g for 30 min. Discarded the supernatant and obtained the pellets of the exosomes.

Isolation of exosomes from serum was performed using Total Exosome Isolation Kit (Life Technologies, Carlsbad, USA) according to the manufacturer’s instruction. The serum volume was fixed at 1000μl for testing exosomal miRNAs by qRT-PCR of samples both from mice and human. Because the blood that can be collected from one mouse is scant, sera of three or four mice were mixed together to achieve the fixed volume of one sample, and each group included 3samples. Firstly, the serum sample was centrifuged at 2000 × g for 30 minutes to remove cells and debris. Then, the clarified serum was transferred to a new tube and add 0.2 volumes of the Total Exosome Isolation reagent, following vortexing and incubating at 4°C for 30 minutes. After incubation, the sample was centrifuged at 10000 × g for 10 minutes at room temperature. Discarded the supernatant, exosomes were contained in the pellet at the bottom.

### Transmission Electron Microscopy (TEM)

Isolated exosomes were resuspended and fixed with 2% paraformaldehyde. A drop of exosome suspensions (approximately10ul) was applied onto a formvar coated copper grid. Then, exosomes were negatively stained with 1% aqueous uranyl acetate for 2 min and wicked off with filter paper. Examination was operated using a FEI Tecnai G2 Spirit Bio TWIN transmission electron microscope (FEI. Hillsboro, USA).

### Nanoparticle tracking analysis

Nanosight NS300 system (Malvern Instruments Ltd, Malvern, United Kingdom) was used to analyze the size distribution and concentration of the isolated exosomes. The exosome preparations were resuspended with 800 ul sterile phosphate saline buﬀer (PBS) and homogenized by vortexing. Data was analyzed by NTA 3.1 Build 3.1.54 software.

### RNA extraction and qRT-PCR

The total RNA of islets and exosomal RNA of islets and serum were extracted using RNeasy Mini Kits and miRNeasy Mini Kit, respectively (QIAGEN [Shanghai] Co. Ltd, Shanghai, China). Exosomes purified from a certain volume of islets culture medium or serum were diluted with 700 μl of Qiazol Lysis Reagent (QIAGEN [Shanghai] Co. Ltd, Shanghai, China) followed by adding 20 fmol synthetic *C. elegans* cel-mir-39 to each sample used for qRT-PCR [36]. Subsequent extraction work was carried out following the manufacturer’s protocols. RNA concentration was measured with a Nanodrop spectrophotometer, ND-2000 (Thermo-Fisher, Waltham, USA).

Expression of miRNA was analyzed using MiR-X miRNA qRT-PCR SYBR Kit and One Step SYBR PrimeScript RT-PCR Kit, respectively (Clontech Laboratories, Inc., Mountain View, USA). All quantitative PCR assays were performed on Step One Plus Real-Time PCR System (Applied Biosystems, Foster City, USA). The levels of *C. elegans* cel-mir-39 spike-in and *Gapdh* was used as endogenous control to normalize miRNA expression in exosomes and mRNA levels in islets, respectively. The Ct values for each gene were normalized to endogenous control, and the relative fold change values were calculated using the ΔΔCt method.

### Microarray analysis

The expression profiles of exosomal miRNA derived from mixed cytokines and STZ treated or control islets were evaluated using Agilent Mouse miRNA Microarray Based on miRBase Release 21.0 (Agilent technologies, Santa Clara, USA) which analyzes 1881 mature miRNAs totally. Firstly, total 100 ng exosomal RNA was labeled by miRNA Complete Labeling and Hyb Kit (Agilent technologies, Santa Clara, USA). Then, each slide was hybridized with Cy3-labeled miRNA in hybridization Oven (Agilent technologies, Santa Clara, USA) at 55°C, 20 rpm for 20 hours. After hybridization, slides were washed in staining dishes (Thermo Shandon, Waltham, USA) with Gene Expression Wash Buffer Kit (Agilent technologies, Santa Clara, USA) and scanned by Agilent Microarray Scanner (Agilent technologies, Santa Clara, USA). Raw data were normalized by Quantile algorithm included in the R package AgiMicroRna [37]. After normalization of the original data, the differential expressed miRNAs were screened using fold-change and Student's t-test statistical method. P<0.05 was considered statistically significant.

### Western blot assay

Exosomes or cells were lysed by RIPA lysis buffer (50 mmol/l Tris [pH 7.4], 150 mmol/l NaCl, 1mg/ml SDS, 0.25% Na deoxycholate, 2% v/v Triton-X100, 1 mM PMSF, 2umol/l leupeptin) on ice. Protein concentration was measured using Pierce BCA Protein Assay Kit (Pierce Biotechnology, Rockford, USA). Western blotting was performed with the indicated antibodies: anti-FAS, anti-DDIT3 (C/EBP homologous protein, CHOP), anti-GAPDH and anti-b TUBULIN (Abcam, Cambridge, United Kingdom); anti-CD63 and anti-CD81 (SBI, Palo Alto, USA). The protein blots were visualized using SuperSignal West Pico Chemiluminescent Substrate (Pierce Biotechnology, Rockford, USA) in ChemiDoc XRS+ system (BioRad, Hercules, CA, USA).

### Statistical analysis

Experimental data was presented as means±SEM of at least three independent experiments. Two-tailed Student’s t tests were applied to assess diﬀerences. *P* values <0.05 were considered statistically significant. All statistical analyses were performed using Statistical Package for Social Science for Windows (SPSS, Version 13.0).

## Supplementary Material

Supplementary Figure

Supplementary Table
