# Supplementary material for: Injury factors alter miRNAs profiles of exosomes derived from islets and circulation
Source: Aging (Albany NY). 2018 Dec 14;10(12):3986–99. doi: 10.18632/aging.101689 (PMC6326691; doi:10.18632/aging.101689)
Supplement: Supplementary Figure [file aging-10-101689-s001.pdf]

SUPPLEMENTARY FIGURE

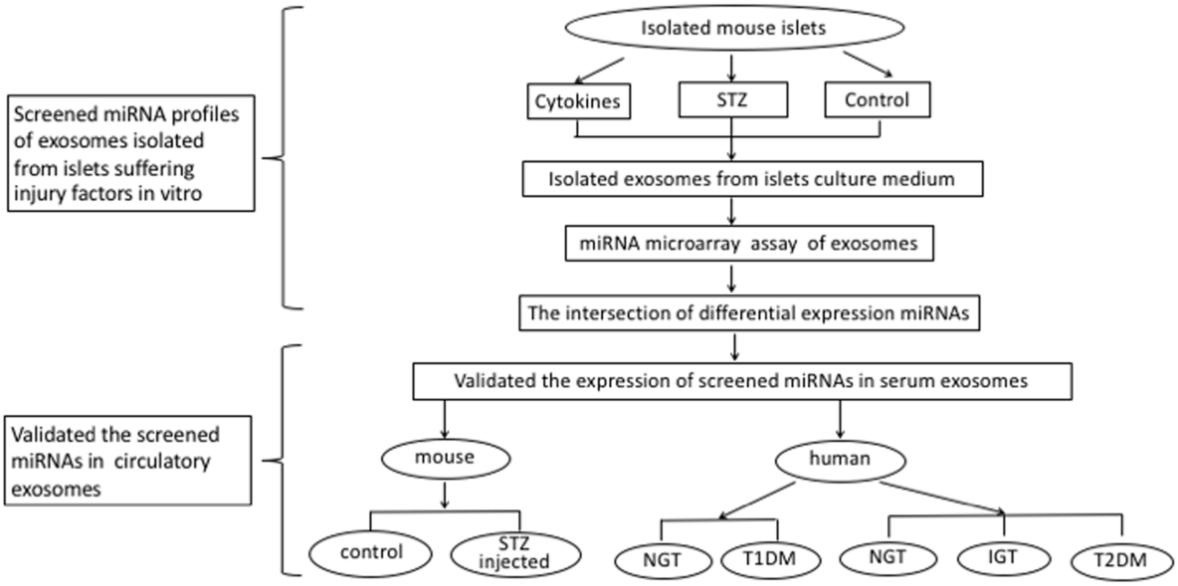

Supplementary Figure S1. The flow diagram of the present experiment. This study aimed to investigate the impact of injury factors on the miRNA profiles of islet exosomes and determine whether circulating exosomal miRNAs would be suitable as biomarkers of islets damage. Firstly, we screened miRNA profiles of exosomes isolated from islets suffering injury factors in vitro. Both cytokines (TNFα, IL-1β and IFNγ cocktail) and streptozocin (STZ) were used to induce islet injury in vitro, respectively. The miRNA expression profiles of exosomes derived from islets suffering different injury factors were screened using microarray assay, and the intersection of the two treatments were selected as further verification indices. Next, we validated the screened miRNAs in circulatory exosomes. We explored whether the screened miRNAs of exosomes derived from islets in vitro could be detected in circulation and considered as novel biomarkers of islets damage, circulating exosomes were isolated from diabetic mouse models and human patients and analyzed by qRT-PCR. NGT: normal glucose tolerance, IGT: impaired glucose tolerance, T1DM: type 1 diabetes mellitus, T2DM: type 2 diabetes mellitus.
