# Supplementary material for: Injury factors alter miRNAs profiles of exosomes derived from islets and circulation
Source: Aging (Albany NY). 2018 Dec 14;10(12):3986–99. doi: 10.18632/aging.101689 (PMC6326691; doi:10.18632/aging.101689)
Supplement: Supplementary Table [file aging-10-101689-s002.pdf]

SUPPLEMENTARY TABLE

Supplementary Table S1. The sequences of miRNAs screened in exosomes of mouse islets suffering injury factors.

miRNAs	RNA sequence
mmu-miR-375-3p	UUUGUUCGUUCGGCUCGCGUGA
hsa-miR-375	UUUGUUCGUUCGGCUCGCGUGA
mmu-miR-129-5p	CUUUUUGCGGUCUGGGCUUGC
hsa-miR-129-5p	CUUUUUGCGGUCUGGGCUUGC
mmu-miR-378a-3p	ACUGGACUUGGAGUCAGAAGG
hsa-miR-378a-3p	ACUGGACUUGGAGUCAGAAGGC
mmu-miR-382-5p	GAAGUUGUUCGUGGUGGAUUCG
hsa-miR-382-5p	GAAGUUGUUCGUGGUGGAUUCG
